# The role of massive demographic databases in intractable illnesses: Denomics for dementia

**DOI:** 10.3934/publichealth.2022043

**Published:** 2022-08-23

**Authors:** Mark M. Stecker, Morgan R. Peltier, Allison B. Reiss

**Affiliations:** 1 Fresno Institute for Neuroscience, Fresno, CA 93730, USA; 2 Department of Psychiatry, Hackensack Meridian Health, Neptune City, NJ 07753, USA; 3 NYU Long Island School of Medicine, Mineola, NY, 11501, USA

**Keywords:** dementia, therapy, dataset, medical record, privacy, environment, exposome

## Abstract

Despite intensive research, effective treatments for many common and devastating diseases are lacking. For example, huge efforts and billions of dollars have been invested in Alzheimer's disease (AD), which affects over 50 million people worldwide. However, there is still no effective drug that can slow or cure AD. This relates, in part, to the absence of an animal model or cellular system that incorporates all the relevant features of the disease. Therefore, large scale studies on human populations and tissues will be key to better understanding dementia and developing methods to prevent or treat it. This is especially difficult because the dementia phenotype can result from many different processes and is likely to be affected by multiple personal and environmental variables. We hypothesize that analyzing massive volumes of demographic data that are currently available and combining this with genomic, proteomic, and metabolomic profiles of AD patients and their families, new insights into pathophysiology and treatment of AD may arise. While this requires much coordination and cooperation among large institutions, the potential for advancement would be life-changing for millions of people. In many ways this represents the next step in the information revolution started by the Human Genome Project.

## 1. Introduction

The process of finding the mechanism for and treating a medical disease varies considerably from disease to disease. For example, diseases related to well-defined genetic abnormalities can be effectively studied with *in vitro* and animal systems prior to human testing because there is precise information regarding their pathophysiology. In the infectious diseases, the situation is slightly different. The process of finding the pathogen and either rendering it inactive or adjusting the host defenses to eliminate it can be studied in many different model systems so that much is understood prior to applying a treatment to humans. However, even in this case, few treatments arise purely *ab initio* from basic mechanistic studies. In fact, many successful treatments have arisen out of the observation of experiments provided by thousands of years of natural medicine.

The case of dementia is much more complex. One reason is that dementia encompasses a number of different diseases with similar phenotypes that develop slowly over a number of years. However, even just one type of dementia, Alzheimer's disease (AD), has proven exceedingly difficult to understand and treat. AD is a progressive, age-related, neurodegenerative disorder that affects >50 million people globally [1] for which there is currently no cure. By 2050, it is estimated that the number of persons with AD worldwide will be above 100 million [2]. Despite billions of dollars of investment, progress has been imperceptible while the need for a leap forward is increasingly urgent. Clinically, AD is characterized by memory loss and a gradual decline in cognitive function eventually leading to incapacity and death, so the consequences to patients and families is incalculable [3] There has been extensive research into the diagnosis and classification of the dementias as well as the molecular changes that may occur in patients and model systems. Despite this, pharmacological therapies have failed in clinical trials and no treatment has significantly modified the trajectory of inexorable decline [4]–[6] Aducanumab, an amyloid-clearing monoclonal antibody, was recently approved by the FDA, but is highly controversial [7].

One reason for the lack of progress in developing AD therapeutics is that AD does not have an abrupt onset and stereotyped evolution. Thus, primary causes are difficult to discern and the individual benefit of any new treatment is difficult to assess. Another obstacle is the difficulty in recruiting AD patients into clinical trials, especially since a portion of these individuals may not have capacity to consent [8] and others may have difficulty travelling to the site performing the trials. This means that patients are often studied after a long period of progressive evolution. Evaluation at this time may not reveal the primary changes that initiated the process of degeneration only the secondary changes that occurred after years of disease activity. Only elucidation of the primary causes can allow the identification of mechanisms and the optimal timing for starting any type of therapy. Thus, we propose here a new approach to finding disease-modifying AD therapy utilizing massive population databases. These types of databases contain longitudinal data from not just the 5–10 thousand patients typically considered in a large medical study, but data on hundreds of millions of people which could be aggregated and analyzed for factors that might influence dementia.

## 2. Utilizing societal resources to understand disease mechanisms

Challenges are the engines that galvanize our society. The space race, the war on cancer, the war on HIV, and the human genome project showed how the process of solving specific and narrowly formulated problems leads to more generalized and often unanticipated improvements in our quality of life [9]–[11] In these cases, society recognized the need and allocated resources for relatively small groups of experts to come up with a solution. However, there are problems with a much greater degree of intricacy and even greater impact that will be solved only if larger sections of our society collaborate to achieve the goal.

Consider the problem of curing AD, which touches so many patients and their families. Companies involved in electronic and in-person commerce such as the pharmaceutical and food industries have massive databases and most medical information is now being entered into an electronic medical record [12],[13]. Governmental organizations are also collecting increasing amounts of data. The technologies required to organize this information while respecting the privacy of individuals and specific corporate interests already exist [14]. Our hypothesis is that by leveraging these technologies, it will be possible to aggregate demographic, genomic, medical, and lifestyle information on millions of people over years to generated hypotheses regarding the most likely pathways contributing to the etiopathology of AD. This information can be melded with existing molecular, genetic and clinical information derived from more detailed studies on smaller populations to accelerate the development of effective treatments. For simplicity, this complex process can be referred to as “denomics”.

**Figure 1.**
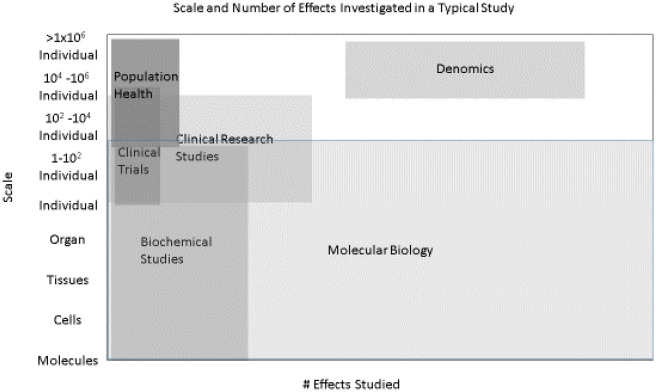
Illustration and comparison of the scale of various research study types.

**Figure 2.**
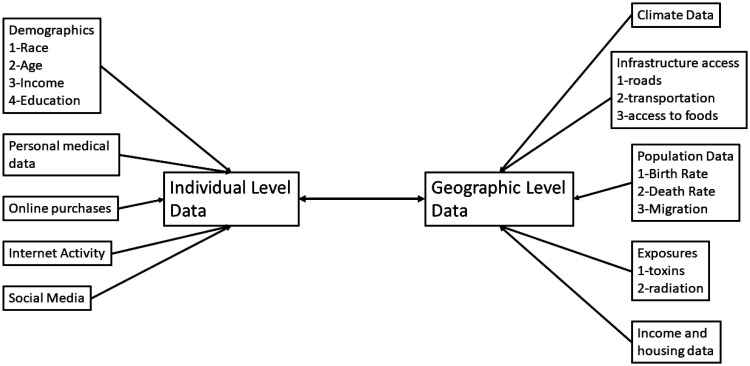
An outline of individual and geographic sources of denomic information. As each of these sources has different levels of sensitivity, different levels of security and permission may be required as discussed in the section on privacy and data security.

As illustrated in Figure 1, there are many different study types in the life sciences. Biochemical studies typically focus on a few carefully selected pathways or molecules with studies at larger scales necessarily studying smaller number of effects. In clinical trials generally there are a very few effects studied although the number of patients may be moderately large. In molecular biology, the number of effects studied can be huge but the number of specific cases studied is often not large. Even if a large number of patients are included in a study, data is often pooled prior to analysis into smaller groups. Denomics addresses studies with large numbers of individual patients and a large number of effects. Although each study type is important, denomics may be optimal for creating specific hypotheses that can be tested by other studies that rely on a priori selection of variables (Figure 2).

## 3. Conquering AD: Bringing together the corporations, the medical community and the pharmaceutical industry

Unraveling the complex etiology of AD requires a new approach and our growing ability to aggregate data from neuroimaging and genomic databases of both healthy and diseased cohorts can provide clues to how genetic and environmental factors affect brain bioenergetics and function. This newfound knowledge can be applied to find the neural pathways and genetic mechanisms underlying AD pathobiology.

Companies have amassed huge databases, often as a byproduct of some discrete commercial goal, that reveal a great deal of information on individuals' behavior and lifestyle [15]–[17]. They know what kind of food we eat, what kind of detergents we use, how we use computers and much, much, more. Wireless companies know how much time we spend in what type of environments. Internet service providers possess data on our sleep habits and even how we type and use language. The challenge is that there is very little incentive to combine these sources, and privacy problems exist with utilizing these datasets for research purposes within commercial companies [18]. The process of aggregating as large a portion of this data as is feasible into a repository that has security, anonymity and privacy as its core tenants is a challenging process that requires action not only for funding, coding and supervision but also governmental regulation. Once created, such a repository could be used to study any disease entity through appropriate statistical testing for correlations between its variables and the disease markers. We hypothesize that this will be a useful step in generating new hypotheses regarding pathophysiology of not only AD but other diseases. In many ways, this process is the modern version of many important medical treatments arising out of observation. One example would be using the observation that people who eat the bark of certain trees have less severe malaria symptoms to develop an effective treatment for this disease. However, when experience with a treatment is short, the disease studied is variable and the complexity of data is high, such as in identifying treatments for Covid-19, there can be much controversy about results that can be resolved only with larger databases containing more detailed information over extended periods of time [19],[20].

Another advantage of this aggregated demographic data (“the denome”) is that with specific patient consent, it can trigger clinician, researcher or vendor contacts reducing the costs for and accelerating the pace at which clinical trials and basic research can be accomplished while also helping to guide patients toward needed clinical resources (Table 1).

**Table 1.** Critical partnerships for creating the demographic dataset for insight into AD contributing factors.

Partner	Role
Individuals	Sharing data, using data, advocating for appropriate data usage, monitoring data usage.
Private companies	Organizing, monitoring, protecting the confidentiality of data.
Advocacy foundations	Collaborates with private companies, individuals and government to refine goals of data collection and optimizing collection mechanisms, data confidentiality.
Government (Federal, state, local)	In conjunction with other groups creates regulations governing the safe use of data and facilitates partnering among different groups.
National Institutes of Health	Funding specific data uses and infrastructure.
Pharmaceutical industry	Funding, integrating clinical trial data, drug discovery, development and marketing.
Universities	Coordinating ideas from many disciplines to refine and enhance the data collection methodology.

Collecting and organizing existing data is important but checking and verifying data is equally important as is adding dementia-specific information that could be done using a crowdsourcing-based technology [21],[22]. This can be supplemented by subjective oral/video histories provided by patients and families telling their stories from which additional information can be abstracted [23],[24].

## 4. The exposome as a critical component

Since AD is largely believed to have a complicated dependence on both genetic and environmental factors, incorporation of environmental considerations into any model seeking to understand AD development is essential [25]–[27]. It is well known that genetic factors influence human cognitive function the aggregation of which is the genome. While many risk loci have been identified for AD, the most widely known is the apolipoprotein E4 genotype, an established risk factor of delayed, sporadic AD [28]. Lifelong, environmental exposures also influence human cognitive function [29],[30], the collection of which is the exposome, a subset of the denome, and are important in determining disease propensity [31]–[33].

The analysis and detection methods for evaluating the exposome mainly involve mass spectrometry (MS), chromatography-MS, liquid chromatography–high resolution MS, electrochemical methods, and fluorescence imaging [34],[35] as well as cell culture and physiologic testing [36]. However, this detailed information must be combined with geographic and demographic information to determine the specific exposures for individuals [37],[38] which are critical to any models. Screening and validating appropriate exposome databases in the context of extensive demographic data, can be used to estimate exposures in patients with AD and lead to specific, novel and testable hypotheses.

## 5. The molecular aspect

In order to move from the conceptual to the pragmatic, the aggregate findings of the exposome, denome and genome must be tested in appropriate living models which may include animal and cell culture systems [39],[40]. These systems can be used to develop and test treatments or prophylaxis for AD. State-of-the-art sequencing technology can identify biomarkers and differentially expressed genes that are affected by environmental toxicants in different genetic backgrounds and then changes in these genes and their corresponding pathways with the application of putative treatments can be documented. Ultimately, this could lead to design of a personalized plan of therapy for each individual AD patient based on precision medical screening as is done for some cancers [41].

## 6. Privacy and data security

In order for denomics to provide useful data that does not interfere with individual privacy, it is important that data at an individual level be entered into a massive database in a manner that makes it impossible to extract the identity of the person associated with the person. However, no matter how secure a database is, there are always ways that it can be compromised. Thus, it is important to allow individuals to control the use of their data. One individual choice might be to include no data use at any level in the database. Another might restrict the use of the data to collections at the level of zip codes, counties or states. The uniqueness of a person's data decreases by having records of large numbers of similar people in the database, greatly reducing the chance of them being re-identified. Completely de-identifying people is problematic, however, because updating data at the individual level would require the presence of individual identifiers. Vendors that collect other data can ask for consent or the consenting process can be undertaken when an individual applies for state identification, driver's license or during completion of forms for the internal revenue service (IRS) among other options. These options are currently used to consent for organ donation or political contributions and so extending these programs to improve public health is also reasonable.

**Figure 3.**
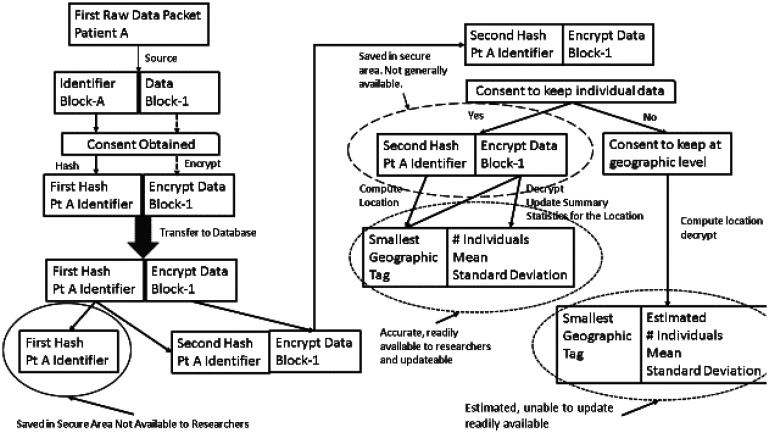
Illustration of a relatively secure database structure that could be used in denomic studies.

Figure 3 illustrates one concept as to how the denomics data flow might be structured to minimize risk of malicious use of the data. Raw data is sent into the system from multiple sources. At the source, all direct identifiers such as name, date of birth, and social security number can be hashed to create a new identifier that is unique but cannot be decoded to yield back the basic identifying information. This hashed ID then labels the data sent to the denomics database so that none of the raw identifier data is ever present in the database and cannot be extracted from the database. This scheme allows updated data to be matched to individuals and updated in the database. This hash is kept in a separate secure area while a second hash of the identifier data is sent along with the data to the main database. At this point, the unique identifiers in the main database are not the same as those generated at the source and so a hacker who might collect the hashed ID's at the source cannot match them to the data in the database unless there is access the hashed ID sent from the source and to the hashing algorithm used to create the main data labels. Access to this data must be kept secure and accessible only by duly authorized personnel onsite with specific access granted by a number of different members on the group supervising the database to prevent hacking.

**Figure 4.**
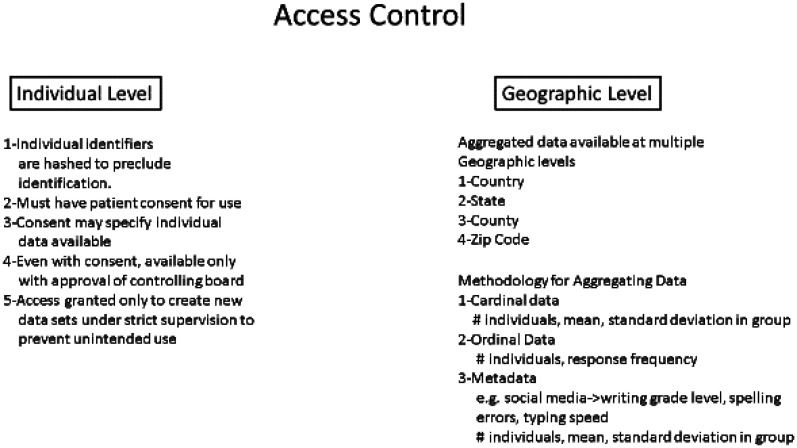
Illustration of access control options for denomic data.

Even with the level of security discussed above, data at the individual level cannot be made publicly available as it may be possible when there is a conjunction of rare diagnoses, demographic or laboratory values to predict with high likelihood that the data came from a specific individual. For example, there may be only a single person in a county with dementia and neurofibromatosis. Thus, access to the individual data even after re-hashing the identifiers must be limited. Thus, individual authorization for various data uses is essential. By entering their basic identifiers, the hash can be created and used to delete or modify the acceptable uses of certain data (Figure 4). Aggregated data however is intrinsically safe as no individual information is available although there is much less power in analyzing data at that level than the individual level. As a result, it will be important to allow different types of access to the database. The public may see data aggregated over sufficiently large geographic regions. However, access to the de-identified individual data would be restricted to regular publication of the geographic based data and to researchers with specific approved research studies that really require this. Access to the de-identified individual data however would be allowed on site only to minimize the chance of a data breach. No access to the original hashed ID would be allowed.

## 7. Challenges of large databases

Beyond the issue of security, there are many problems with large databases. First, errors are inevitable and need to be minimized. This can be done by regular data checking as each column of data is added to the database it should be documented and have parameters for what constitutes good data. Bad data can be marked appropriately. Checksums can be implemented to detect errors during data transmission and processing. During the lifespan of the database, there may be repeated uploads from the same source at different times to capture new data or uploads from different sources that contain similar data. Identifying inconsistencies in this data is also important in assuring data integrity. Inaccurate data is harder to find but criteria for mean and variance can be set to flag suspect data for closer inspection.

Other problems relate to the difficulty of doing true multivariate analyses in a large dataset. There needs to be the ability to perform univariate tests of correlation in an initial stage to select potential variables for analysis as well as complex multivariable analyses. However, the biggest issue is that it is difficult to determine statistical significance when the number of variables greatly exceeds the number of cases (individuals or geographic units). For example, if the analysis was restricted to data aggregated over counties in the US there are only 3200+ cases while there can be many more variables. Standard protocols along with data simulations need to be created for optimal analysis that show the strength and limitations of each technique.

## 8. Conclusions

Given the overall projected increase in life expectancy across the globe, massive efforts are warranted to identify modifiable risk factors and to find new neuroprotective medicines to treat/prevent AD. The scope of this project is immense, but the rewards will go far beyond the direct benefit of effectively treating a devastating illness. It will serve as a template for managing other disorders that have been intractable to date. However, its greatest accomplishment would be bringing together a large community of politicians, businesspeople, ethicists, researchers, clinicians, and technology specialists. The power of such a group to focus on and improve the problems of our society would be amazing.

In order to make tangible progress, we propose a major public health initiative to combat dementia involving participation of all sectors of our society [42]. It should not be designed only by experts in dementia, but should also include representatives with many different skillsets and from every facet of society. Gathering support of large segments of the public would join everyone in working together for the good of all.
